# Supplementary material for: Pan-cancer pervasive upregulation of 3′ UTR splicing drives tumourigenesis
Source: Nat Cell Biol. 2022 May 26;24(6):928–39. doi: 10.1038/s41556-022-00913-z (PMC9203280; doi:10.1038/s41556-022-00913-z)
Supplement: Supplementary file 1 — Supplementary Notes. [file 41556_2022_913_MOESM1_ESM.pdf]

nature portfolio

https://doi.org/10.1038/s41556-022-00913-z

Supplementary information

Pan-cancer pervasive upregulation of 3′ UTR splicing drives tumourigenesis

In the format provided by the authors and unedited

Supplementary Notes

The identified 3’UTR splicing events were highly consistent across different datasets (related to Fig. 1d, 3c, Extended Data Fig. 1a,b and 3a)

To check the reproducibility of the identified 3’UTR splicing events across different datasets, we first overlapped the c3USPs from TCGA-normal and GTEx for each tissue. As the number of overlapped events was influenced by the number of identified c3USPs, for example the breast (TCGA-BRCA) cohort with the highest number of c3USPs yielded the highest number of overlapped events, we used the percentage of overlapping TCGA- and GTEx-derived c3USPs as an indication of consistency. The percentage of events from overlapping the same tissue (average ~90%) was higher than across different tissues (average ~83%) between TCGA-normal and GTEx (Extended Data Fig. 1a). We applied this analysis to the 3USPs and found no significant differences between the overlaps of the same tissue and across different tissues (Extended Data Fig. 1b). Furthermore, both 3USPs and c3USPs showed a significantly higher percentage of overlapping events compared to the backgrounds (Extended Data Fig. 1b). In brief, the backgrounds were generated by taking the union of all the identified 3USPs from nine TCGA cohorts and eight GTEx tissues. Next, for each tissue, the same number of events with respect to the identified 3USPs or c3USPs was selected from this union set. We repeated this 100 times and used the average numbers to perform the overlaps. We also checked the percentages of 3’UTR splicing events identified in TCGA-normal, TCGA-tumor and GTEx, which were supported by PacBio data. The majority of c3USPs (~50%) in all three datasets were supported by PacBio data compared to only ~10% of 3USPs (Fig. 1d). We also showed that there was more than 90% of overlap for the c3UPSs identified from TCGA-LIHC and PLANet datasets (Fig. 3c). Due to the small sample size of the in-house dataset (n=4), we identified only 3USPs, overlapped them with those identified in the TCGA-LIHC dataset (n=371) for comparative analysis and found that 82.5% of events were shared (Extended Data Fig. 3a).

3’UTR splicing may not trigger nonsense-mediated mRNA decay (NMD) and may mitigate Staufen-mediated mRNA decay (SMD) (related to Fig. 1e,f and Extended Data Fig. 1f-l)

As splicing downstream of stop codons (typically >50nt) is known to trigger NMD, which results in the degradation of the spliced transcript[1], we grouped 3USPs and c3USPs based on their splicing levels and

analyzed their distances from the stop codon. We found that the events with higher splicing levels tended to be closer to the stop codon (Fig. 1e and Extended Data Fig. 1f-j). In particular, TCGA-tumor- and TCGA-normal-derived c3USPs had a median distance of 40nt and 41nt from the stop codon respectively, indicating that the majority were unlikely to be NMD targets. Furthermore, splicing levels were significantly higher in 3'UTR introns containing Alu repeat elements (Fig. 1f and Extended Data Fig. 1k,l), suggesting that the removal of Alu-rich RNA fragments via 3'UTR splicing may mitigate SMD[2].

Survival analysis to identify prognostic c3USPs (related to Fig. 1i and Extended Data Fig. 3e)

We performed univariate Cox proportional hazards regression analysis and Kaplan-Meier analysis to correlate the splicing levels of each c3USP with overall patient survival in each cancer type ($p<0.05$). The ratios of unfavorable and favorable prognostic c3USPs across different cancer types derived from these two methods were similar.

Dysregulated c3USPs identified from the PLANet and TCGA-LIHC datasets are significantly correlated (related to Extended Data Fig. 3b)

For each c3USP event identified in both the TCGA-LIHC and PLANet tumor samples (n=714), we first calculated the proportion of tumor samples that were over- and under-spliced. Next, the difference in proportion between the over- and under-spliced samples was used to evaluate the dysregulation. The difference in proportion derived from the PLANet and TCGA-LIHC datasets were significantly correlated ($r=0.465$, $p < 3.4e-49$). We also identified significantly dysregulated events using the following criteria: (1) FDR <0.1 when comparing the SPLs between tumor and normal; (2) difference in proportion between over-spliced and under-spliced tumor samples >5%. In total, 218 and 118 significantly dysregulated c3USPs were identified from PLANet and TCGA-LIHC respectively, while 57 c3USPs were significant in both datasets. The correlation of these 57 events between the two datasets was even more evident ($r=0.733$, $p < 9e-11$).

Specificity and stability of the 3'UTR splice site-targeting ASOs (related to Fig. 3e-h, 5g, Extended Data Fig. 3c-f, 4, 8g-i, Supplementary Table 8)

The ASOs designed to block the *CTNNB1* 3'UTR 5' splice site contained two chemical modifications: phosphorothioate bonds and 2'-O-Methyl RNA bases. These have been shown to enhance binding affinity and specificity as well as inhibit nuclease degradation of the target transcript[3]. We showed that ASO-SS downregulated only the 3'SP transcript but not the 3'FL transcript, indicating that it was acting to specifically block splicing of the 3'UTR.

Effect of ASO-mediated blocking of 3'UTR splice sites (related to Fig. 3f and Extended Data Fig. 3d)

The *CTNNB1* band marked by the arrowhead (◄) is a second 3'UTR splice variant (3'SP2), which is not a significantly dysregulated splicing event in HCC, therefore it was not included in further experiments. Additionally, unlike *CHEK1*, *CTNNB1* and *THUMPD1*, no PCR bands were observed for the full length 3'UTR transcript (3'FL) for *MAPK1* and *WDR55*. This could be due to the large intron sizes (4,954nt for MAPK1 and 1,578nt for *WDR55*), and the low abundance of these 3'FL transcripts relative to their respective 3'SP.

Specificity of the 3'UTR-targeting siRNAs (related to Fig. 5b-g, Extended Data Fig. 6b-g, 7, 8j-m, Supplementary Table 6)

The BLAST query of the si-3'FL and si-3'SP sequences showed that both siRNA designs were highly specific, thus the downregulation of the 3'FL transcript by si-3'SP could be a secondary rather than an off-target effect.

References

1. Nagy, E. & Maquat, L.E. A rule for termination-codon position within intron-containing genes: when nonsense affects RNA abundance. *Trends Biochem. Sci.* **23**, 198-199 (1998).
2. Park, E. & Maquat, L.E. Staufen-mediated mRNA decay. *Wiley Interdiscip Rev RNA* **4**, 423-435 (2013).
3. Roberts, T.C., Langer, R. & Wood, M.J.A. Advances in oligonucleotide drug delivery. *Nat. Rev. Drug Discov.* (2020).
