# Supplementary material for: Pan-cancer pervasive upregulation of 3′ UTR splicing drives tumourigenesis
Source: Nat Cell Biol. 2022 May 26;24(6):928–39. doi: 10.1038/s41556-022-00913-z (PMC9203280; doi:10.1038/s41556-022-00913-z)
Supplement: Supplementary file 2 — Reporting Summary [file 41556_2022_913_MOESM2_ESM.pdf]

nature portfolio

Corresponding author(s): Yvonne Tay

Last updated by author(s): Feb 22, 2022

Reporting Summary

Nature Portfolio wishes to improve the reproducibility of the work that we publish. This form provides structure for consistency and transparency in reporting. For further information on Nature Portfolio policies, see our Editorial Policies and the Editorial Policy Checklist.

Statistics

For all statistical analyses, confirm that the following items are present in the figure legend, table legend, main text, or Methods section.

n/a	Confirmed	
☐	☒	The exact sample size (n) for each experimental group/condition, given as a discrete number and unit of measurement
☐	☒	A statement on whether measurements were taken from distinct samples or whether the same sample was measured repeatedly
☐	☒	The statistical test(s) used AND whether they are one- or two-sided<br>*Only common tests should be described solely by name; describe more complex techniques in the Methods section.*
☐	☒	A description of all covariates tested
☐	☒	A description of any assumptions or corrections, such as tests of normality and adjustment for multiple comparisons
☐	☒	A full description of the statistical parameters including central tendency (e.g. means) or other basic estimates (e.g. regression coefficient) AND variation (e.g. standard deviation) or associated estimates of uncertainty (e.g. confidence intervals)
☐	☒	For null hypothesis testing, the test statistic (e.g. F, t, r) with confidence intervals, effect sizes, degrees of freedom and P value noted<br>*Give P values as exact values whenever suitable.*
☒	☐	For Bayesian analysis, information on the choice of priors and Markov chain Monte Carlo settings
☒	☐	For hierarchical and complex designs, identification of the appropriate level for tests and full reporting of outcomes
☒	☐	Estimates of effect sizes (e.g. Cohen's d, Pearson's r), indicating how they were calculated

Our web collection on statistics for biologists contains articles on many of the points above.

Software and code

Policy information about availability of computer code

Data collection	CellSens (v1.15) was used for imaging soft agar assays.<br>Fluoview (v3.0) was used for confocal microscope imaging for RNA-FISH.
Data analysis	The custom codes for 3'UTR splicing events identification, filtering, analysis of the features , have been made publicly available on GitHub at https://github.com/christear/RNASeq3USP<br>Additionally, STAR (v2.52a), BEDTools (v2.29), featureCounts (v1.6.1), SAMtools (v1.8), Perl (v5.26) and R (v4.1.2) were used.<br>ImageJ (v1.51j8) was used for anchorage-independent growth and RNA-FISH image processing and analysis.<br>CellSens (v1.15) was used for cell migration analysis.

For manuscripts utilizing custom algorithms or software that are central to the research but not yet described in published literature, software must be made available to editors and reviewers. We strongly encourage code deposition in a community repository (e.g. GitHub). See the Nature Portfolio guidelines for submitting code & software for further information.

Data

Policy information about availability of data

All manuscripts must include a data availability statement. This statement should provide the following information, where applicable:

- Accession codes, unique identifiers, or web links for publicly available datasets
- A description of any restrictions on data availability
- For clinical datasets or third party data, please ensure that the statement adheres to our policy

All c3USPs across 10 TCGA cancer types and their corresponding normal tissues, as well as the patterns of splicing level, can be found on the SpUR database: http://www.cbrc.kaust.edu.sa/spur/home. It also provides a function to query the association between 3'UTR splicing levels and prognosis in each cancer type.

RNA-seq and clinical data of 10 cancer types in TCGA (The Cancer Genome Atlas) were downloaded from the dbGaP TCGA repository.
RNA-seq data of the corresponding normal tissues were downloaded from the dbGaP TCGA repository and the GTEx (The Genotype-Tissue Expression) portal.
RNA-seq data of the in-house four HCC matched pairs and 55 AML and healthy control samples analyzed in this study have been deposited in Sequence Read Archive (SRA, https://www.ncbi.nlm.nih.gov/sra) with the accession number PRJNA602213.
The PLANet RNA-seq dataset of 211 samples was deposited in EGA.
RNA-sequencing data from PacBio used in this study are released on its official website and can be downloaded from the links below:
http://datasets.pacb.com.s3.amazonaws.com/2014/Iso-seq_Human_Tissues/list.html http://datasets.pacb.com.s3.amazonaws.com/2013/IsoSeqHumanMCF7Transcriptome/list.html
The genome and gene annotation used for the alignment were downloaded from GENCODE (https://www.gencodegenes.org).

Field-specific reporting

Please select the one below that is the best fit for your research. If you are not sure, read the appropriate sections before making your selection.

☒ Life sciences ☐ Behavioural & social sciences ☐ Ecological, evolutionary & environmental sciences

For a reference copy of the document with all sections, see nature.com/documents/nr-reporting-summary-flat.pdf

Life sciences study design

All studies must disclose on these points even when the disclosure is negative.

Sample size	No statistical methods were used to predetermine sample sizes. The 10 cancer cohorts used in this study were selected based on them having sufficient adjacent normal samples (>30). Eight tissues from GTEx were also included in this study since LUAD and LUSC shared the same normal control tissues, and HNSC did not have a good corresponding normal controls. As the study mainly focused on HCC, an additional two datasets of HCC samples, including one with a large sample size (PLANet dataset with 211 samples) and one with small sample size (in-house dataset with 4 matched pairs) were used to further confirm the findings based on the public dataset. To investigate the heterogeneities of 3'UTR splicing events across cancers, we also included a dataset from 55 AML patient samples (34 AML vs. 21 healthy controls). The datasets and number of samples selected were sufficiently large to show statistically significant differences. For biological experiments, sample sizes were selected based on similarly published research.
Data exclusions	80 RNA samples from GTEx were excluded due to low sequencing depth (total number of splicing junctions < 1000). Sample numbers before and after exclusion are shown in Supplementary Table 1. No data were excluded for the biological experiments.
Replication	All experiments were replicated at least three times by two or more investigators. All repeats were reproducible.
Randomization	For computational analyses, samples were grouped by different tissues or cancer types and comparisons between tumors and their corresponding normal tissues were performed in each dataset.<br>Randomization was applied to all in vivo experiments but not in vitro experiments as it was not necessary. For biological experiments, at least two non-targeting/ scrambled control was used in all knockdown and ASO experiments for normalization and the identification of significant gene expression and splicing changes. The relevant empty vector was used as a within-batch control for all overexpression and luciferase studies.
Blinding	No blinding was performed for the computational analyses as these were performed using unbiased software programs or algorithms.<br>Blinding was applied to the data collection of at least one set of each experiment except for RNA-FISH due to the experimental technicality and license requirement for confocal microscopy.

Reporting for specific materials, systems and methods

We require information from authors about some types of materials, experimental systems and methods used in many studies. Here, indicate whether each material, system or method listed is relevant to your study. If you are not sure if a list item applies to your research, read the appropriate section before selecting a response.

Materials & experimental systems

n/a	Involved in the study	
☐	☒	Antibodies
☐	☒	Eukaryotic cell lines
☒	☐	Palaeontology and archaeology
☐	☒	Animals and other organisms
☐	☒	Human research participants
☒	☐	Clinical data
☒	☐	Dual use research of concern

Methods

n/a	Involved in the study	
☒	☐	ChIP-seq
☒	☐	Flow cytometry
☒	☐	MRI-based neuroimaging

Antibodies

Antibodies used	Rabbit monoclonal Recombinant Anti-Cdk2 antibody [E304]; Abcam; Cat# ab32147; Lot# GR292523-12; 1:2,000

Antibodies used

Rabbit monoclonal Recombinant Anti-c-Myc antibody [Y69]; Abcam; Cat# ab32072; Lot# GR3232703-14; 1:2,000
Rabbit polyclonal Anti-THUMPD1 antibody; Abcam; Cat# ab199850; Lot# GR3176375-2; 1:2,000
Rabbit polyclonal Anti-U2AF65 antibody; Abcam; Cat# ab37530; Lot# GR3221592-4; 1:2,000
Rabbit monoclonal Axin2 (76G6); Cell Signaling; Cat# 2151; Lot# 10/2019-2; 1:1,000
Rabbit monoclonal Cyclin E1 (D7T3U); Cell Signaling; Cat# 20808; Lot# 04/2019-3; 1:2,000
Rabbit monoclonal CDK4 (D9G3E); Cell Signaling; Cat# 12790; Lot# 10/2017-4; 1:2,000
Mouse monoclonal CDK6 (DCS83); Cell Signaling; Cat# 3136; Lot# 06/2018-2; 1:2,000
Mouse monoclonal Chk1 (2G1D5); Cell Signaling; Cat# 2360; Lot# 09/2018-3; 1:2,000
Rabbit monoclonal β-Catenin (D10A8) XP®; Cell Signaling; Cat# 8480; Lot# 09/2018-5; 1:2,000
Rabbit monoclonal GAPDH (D16H11) XP®; Cell Signaling; Cat# 5174; Lot# 10/2017-7; 1:10,000
Rabbit monoclonal HA-Tag (C29F4); Cell Signaling; Cat# 3724; Lot# 06/2019-9; 1:1,000
Rabbit monoclonal hnRNP C1/C2 (D6S3N); Cell Signaling; Cat# 91327; Lot# 07/2019-1; 1:1,000
Rabbit monoclonal p44/42 MAPK (Erk1/2) (137F5); Cell Signaling; Cat# 4695; Lot# 11/2018-21; 1:5,000
Rabbit monoclonal SF3B1 (D7L5T); Cell Signaling; Cat# 14434; Lot# 05/2019-1; 1:2,000
Rabbit monoclonal TDP43 (G400); Cell Signaling; Cat# 3448; Lot# 01/2020-2; 1:1,000
Rabbit monoclonal TCF1/TCF7 (C63D9); Cell Signaling; Cat# 2203; Lot# 05/2019-8; 1:1,000
normal mouse IgG; Santa Cruz; Cat# sc-2025; Lot# F1818; 3μg for IP
Mouse monoclonal Anti-SAP 155 Antibody (B-3); Santa Cruz; Cat# sc-514655; Lot# B2621; 3μg for IP
Mouse monoclonal Anti-SF2/ASF Antibody (3G268); Santa Cruz; Cat# sc-73026; Lot# J2717; 1:5,000 for WB, 3μg for IP
Mouse monoclonal Anti-U2AF65 Antibody (MC3); Santa Cruz; Cat# sc-53942; Lot# C1521 ; 3μg for IP
Mouse monoclonal Anti-U1 snRNP 70 Antibody (C-3); Santa Cruz; Cat# sc-390899; Lot# G1219; 3μg for IP
Mouse monoclonal Anti-WDR55 Antibody (A-5); Santa Cruz; Cat# sc-514225; Lot# D2518; 1:1,000

Validation

Rabbit monoclonal Recombinant Anti-Cdk2 antibody [E304]
Knockout validated in HAP1 cells for WB
https://www.abcam.com/cdk2-antibody-e304-ab32147.html

Rabbit monoclonal Recombinant Anti-c-Myc antibody [Y69]
Knockout validated in HEK-293T cells for WB
https://www.abcam.com/c-myc-antibody-y69-ab32072.html

Rabbit polyclonal Anti-THUMPD1 antibody
Validated using HeLa, 293T, Jurkat cell lysates for WB
https://www.abcam.com/thumpd1-antibody-ab199850.html

Rabbit polyclonal Anti-U2AF65 antibody
Validated using HeLa, Jurkat, A-431, HEK-293, HepG2, MCF-7, SHSY-5Y, U2OS cell lysates for WB
https://www.abcam.com/u2af65-antibody-ab37530.html

Rabbit monoclonal Axin2 (76G6)
Validated using HCT15 and SW620 cel lysates for WB
https://www.cellsignal.com/products/primary-antibodies/axin2-76g6-rabbit-mab/2151

Rabbit monoclonal Cyclin E1 (D7T3U)
Validated using HT-29 cell lysates +/- aphidicolin treatment
https://www.cellsignal.com/products/primary-antibodies/cyclin-e1-d7t3u-rabbit-mab/20808

Rabbit monoclonal CDK4 (D9G3E)
Validated using Jurkat, HeLa, MCF7 and COS-7 cell lysates for WB
https://www.cellsignal.com/products/primary-antibodies/cdk4-d9g3e-rabbit-mab/12790

Mouse monoclonal CDK6 (DCS83)
Validated using HeLa, IM-CD-3, C6 cell lysates for WB
https://www.cellsignal.com/products/primary-antibodies/cdk6-dcs83-mouse-mab/3136

Mouse monoclonal Chk1 (2G1D5)
Knockdown validated in HeLa cells for WB
https://www.cellsignal.com/products/primary-antibodies/chk1-2g1d5-mouse-mab/2360

Rabbit monoclonal β-Catenin (D10A8) XP®
Validated using HeLa, 293T, NIH3T3, C6 cell lysates for WB
https://www.cellsignal.com/products/primary-antibodies/b-catenin-d10a8-xp-rabbit-mab/8480

Rabbit monoclonal GAPDH (D16H11) XP®
Validated using HeLa, NIH3T3, C6, COS-7 cell lysates for WB
https://www.cellsignal.com/products/primary-antibodies/gapdh-d16h11-xp-rabbit-mab/5174

Rabbit monoclonal HA-Tag (C29F4)
Validated using untransfected vs. HA-FoxO4 or HA-Akt3 transfected HeLa cell lysates for WB
https://www.cellsignal.com/products/primary-antibodies/ha-tag-c29f4-rabbit-mab/3724

Rabbit monoclonal hnRNP C1/C2 (D6S3N)
Validated using HL60, MOLT4, IMR32, COS7 cell lysates for WB
https://www.cellsignal.com/products/primary-antibodies/hnrnp-c1-c2-d6s3n-rabbit-mab/91327

Rabbit monoclonal p44/42 MAPK (Erk1/2) (137F5)

Knockdown validated in HEK-293 cells for WB
https://www.cellsignal.com/products/primary-antibodies/p44-42-mapk-erk1-2-137f5-rabbit-mab/4695

Rabbit monoclonal SF3B1 (D7L5T)
Validated using PANC-1, HeLa, 3T3, H-e-II-E cell lysates for WB
https://www.cellsignal.com/products/primary-antibodies/sf3b1-d7l5t-rabbit-mab/14434

Rabbit monoclonal TDP43 (G400)
Validated using HeLa and rat brain cell lysates for WB
https://www.cellsignal.com/products/primary-antibodies/tdp43-g400-antibody/3448

Rabbit monoclonal TCF1/TCF7 (C63D9)
Validated using HT-29, Colo201, Jurkat and mouse thymocytes cell lysates for WB
https://www.cellsignal.com/products/primary-antibodies/tcf1-tcf7-c63d9-rabbit-mab/2203

normal mouse IgG
No validation information

Mouse monoclonal Anti-SAP 155 Antibody (B-3)
Validated using AMJ2-C8, A549, Raji, WEHI-231, Cak-1, BYDP cell lysates for WB
https://www.scbt.com/p/sap-155-antibody-b-3
Validated using Hep3B cell lysate for IP in lab

Mouse monoclonal Anti-SF2/ASF Antibody (3G268)
Validated using A-431, LADMAC, F9, C6, H19-7/IGF-IR cell lysates for WB
https://www.scbt.com/p/sf2-asf-antibody-3g268
Validated using Hep3B cell lysate for IP in lab

Mouse monoclonal Anti-U2AF65 Antibody (MC3)
Validated using HeLa, HEK293, Jurkat, SK-N-MC cell lysates for WB
https://www.scbt.com/p/u2af65-antibody-mc3
Validated using Hep3B cell lysate for IP in lab

Mouse monoclonal Anti-U1 snRNP 70 Antibody (C-3)
Validated using HepG2, Jurkat, RAW 264.7 cell lysates for WB
https://www.scbt.com/p/u1-snrnp-70-antibody-c-3
Validated using Hep3B cell lysate for IP in lab

Mouse monoclonal Anti-WDR55 Antibody (A-5)
Validated using K-562, SUP-T1, HL-60 cell lysates for WB
https://www.scbt.com/p/wdr55-antibody-a-5

Eukaryotic cell lines

Policy information about cell lines

Cell line source(s)	THLE-2 (ATCC), Hep3B (ATCC), HepG2 (ATCC), SNU398 (ATCC), DLD-1 (Horizon Discovery)
Authentication	All cell lines were authenticated by STR profiling by the suppliers when purchased (COAs available).
Mycoplasma contamination	All cell lines were routinely tested for mycoplasma contamination (every 3 months) and all tested negative.
Commonly misidentified lines (See ICLAC register)	No commonly misidentified cell lines were used in this study.

Animals and other organisms

Policy information about studies involving animals; ARRIVE guidelines recommended for reporting animal research

Laboratory animals	CrTac:NCr-Foxn1<nu> (NCr nude) mice, female, 4-6 weeks old purchased from Invivos<br>The mice were housed in the following conditions: 23-24 °C, 44-58% humidity, 12 h/ 12 h dark/ light cycle (7 pm-7 am/ 7 am-7 pm).
Wild animals	No animals were used in this study.
Field-collected samples	No field-collected samples were used in this study.
Ethics oversight	All mouse work was performed in accordance to the NUS Institutional Animal Care and Use Committee (IACUC) guidelines.

Note that full information on the approval of the study protocol must also be provided in the manuscript.

Human research participants

Policy information about studies involving human research participants

Population characteristics	Clinical information on the TCGA patients used in this study is available via the Genomic Data Commons (GDC) at https://portal.gdc.cancer.gov/. Information on population characteristics of the patients from which the in-house HCC, AML and PLANet samples were derived will be available before publication.
Recruitment	The patients in the PLANet study were recruited under a Translational and Clinical Research (TCR) Flagship Programme: Precision Medicine in Liver Cancer across an Asia Pacific NETwork (PLANet), funded by the Singapore National Medical Research Council (NMRC) programme. A total of 46 patients recruited from Singapore (National Cancer Centre Singapore, Singapore General Hospital, and National University Hospital), Thailand (National Cancer Institute Thailand) and Malaysia (University of Malaya Medical Centre) under the PLANet study were included in this study. PLANet recruited treatment-naïve patient with early stage liver cancer based on AASLD imaging criteria and required the patients to have no extra-hepatic metastasis (defined as lymph node <2 cm, lung modules < 1 cm, farther lymph nodes < 2 cm) with R0 or R1 resection and Child-Pugh ≤ 7 points without clinical ascites. Tumour (T), adjacent non-tumour liver tissue (N) and peripheral blood (P) were collected from these patients and subsequently processed for whole genome sequencing and RNAseq. There are no self-selection bias or other biases that may be present.<br><br>The AML patients recruited were consecutive AML patients presented to NUH who consented to have their samples stored for research. For normals, these are patient undergoing total knee replacements who consented. There is no specific inclusion criteria that will lead to bias.
Ethics oversight	The human studies were approved by the following Institutional Review Boards: the Domain Specific Review Board (DSRB) under the National Healthcare Group (NHG) in Singapore, the Central Institution Review Board (CIRB) of SingHealth, of which all National Cancer Center Singapore, Singapore General Hospital and National University Hospital were constituent members (CIRB Ref: 2016/2626 and 2018/2112), Medical Research Ethics Committee of UMMC (MREC ID NO: 201713-4729) and Research Committee of National Cancer Institute Thailand (Project Number: 174_2017C_OUT504). Each patient gave informed written consent.

Note that full information on the approval of the study protocol must also be provided in the manuscript.
