# Supplementary material for: Pan-cancer pervasive upregulation of 3′ UTR splicing drives tumourigenesis
Source: Nat Cell Biol. 2022 May 26;24(6):928–39. doi: 10.1038/s41556-022-00913-z (PMC9203280; doi:10.1038/s41556-022-00913-z)

All sections presented in the figures are boxed.

Extended Data Fig. 6c unprocessed blot

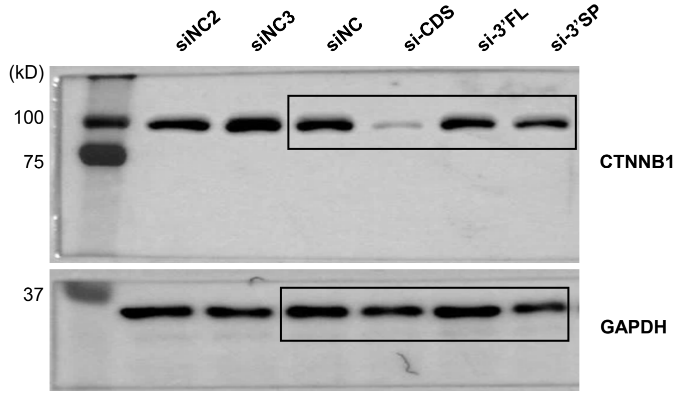

Extended Data Fig. 6g unprocessed blot

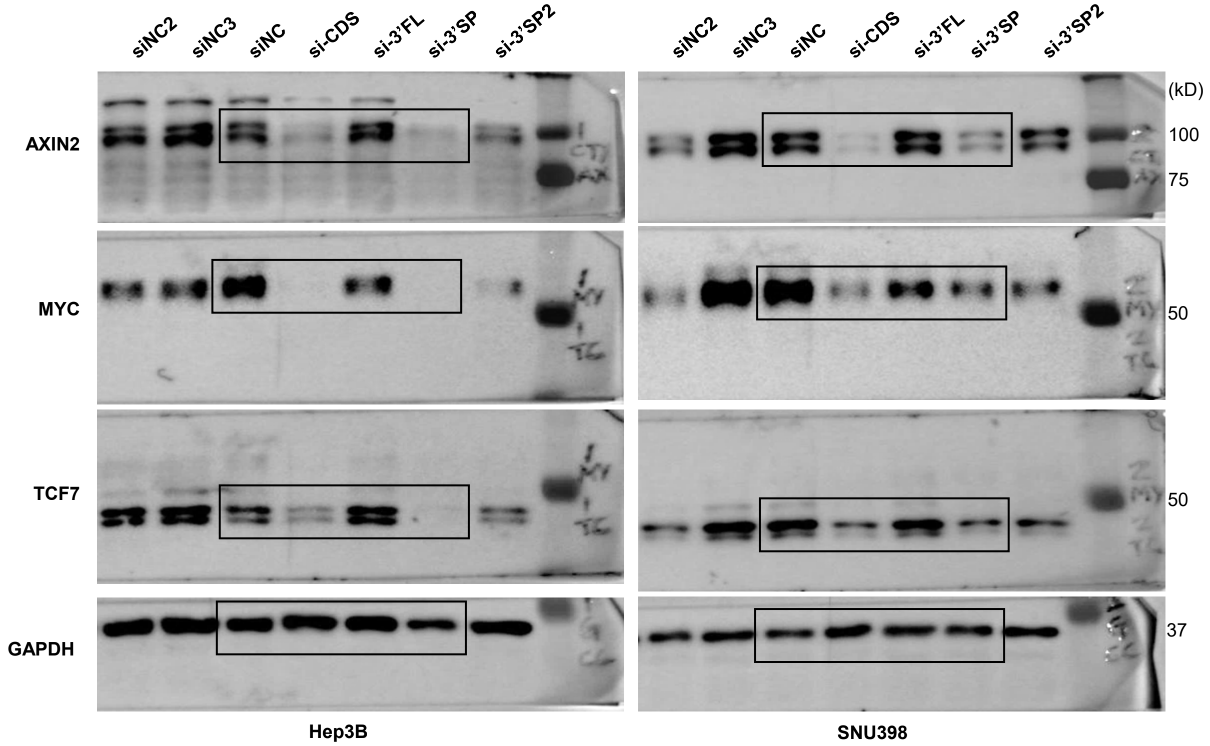

Supplement: Source Data Extended Data Fig. 6 — Unprocessed western blots/gels. [file 41556_2022_913_MOESM26_ESM.pdf]
